# Supplementary material for: Deletion of wfs1 Impairs Oligodendrocyte Precursor Cells Dorsal Distribution and Myelination Through the wfs1-hmgcs1 Axis in Zebrafish
Source: Biology (Basel). 2026 Aug 16;15(16):1402. doi: 10.3390/biology15161402 (PMC13510151; doi:10.3390/biology15161402)
Supplement: Supplementary file 1 [file biology-15-01402-s001.zip › File S1-Transcriptome sequencing data and differentially expressed genes (DEGs) in wfs1-- zebrafish at 5 dpf/2.KEGG/wfs1KOvsWT/UP/src/dre03015.html]

dre03015


- dre:321035

- Up regulated genes

321035\_1(2.36627873946462)

- dre:559392

- Up regulated genes

559392\_1(5.40322756218621)
- dre:562282

- Up regulated genes

562282(1.6446353235091)

- dre:321035

- Up regulated genes

321035\_1(2.36627873946462)

- dre:324267

- Up regulated genes

324267\_1(6.83370155064563)

- dre:100002680

- Up regulated genes

100002680\_2(2.08900246284662)

- dre:100005893

- Up regulated genes

100005893(2.69284130859211)

- dre:393693

- Up regulated genes

393693\_1(8.42626553180885)

- dre:386630

- Up regulated genes

386630\_1(7.78558729858034)

Close
